# Integrated Metabolomic and Lipidomic Analysis Reveals the Neuroprotective Mechanisms of Bushen Tiansui Formula in an A*β*1-42-Induced Rat Model of Alzheimer's Disease

**DOI:** 10.1155/2020/5243453

**Published:** 2020-06-19

**Authors:** Min Yi, Chunhu Zhang, Zheyu Zhang, Pengji Yi, Panpan Xu, Jianhua Huang, Weijun Peng

**Affiliations:** ^1^Department of Integrated Traditional Chinese & Western Medicine, The Second Xiangya Hospital, Central South University, Changsha, Hunan 410011, China; ^2^Department of Integrated Traditional Chinese and Western Medicine, Xiangya Hospital, Central South University, Changsha, Hunan 410008, China; ^3^Hunan Academy of Chinese Medicine, Changsha 410013, China

## Abstract

Bushen Tiansui Formula (BSTSF) is a traditional Chinese medicine prescription. It has been widely applied to treat Alzheimer's disease (AD) in the clinic; however, the mechanisms underlying its effects remain largely unknown. In this study, we used a rat AD model to study the effects of BSTSF on cognitive performance, and UPLC-MS/MS-based metabolomic and lipidomic analysis was further performed to identify significantly altered metabolites in the cerebral cortices of AD rats and determine the effects of BSTSF on the metabolomic and lipidomic profiles in the cerebral cortices of these animals. The results revealed that the levels of 47 metabolites and 30 lipids primarily associated with sphingolipid metabolism, glycerophospholipid metabolism, and linoleic acid metabolism were significantly changed in the cerebral cortices of AD rats. Among the altered lipids, ceramides, phosphatidylethanolamines, lysophosphatidylethanolamines, phosphatidylcholines, lysophosphatidylcholines, phosphatidylserines, sphingomyelins, and phosphatidylglycerols showed robust changes. Moreover, 34 differential endogenous metabolites and 21 lipids, of which the levels were mostly improved in the BSTSF treatment group, were identified as potential therapeutic targets of BSTSF against AD. Our results suggest that lipid metabolism is highly dysregulated in the cerebral cortices of AD rats, and BSTSF may exert its neuroprotective mechanisms by restoring metabolic balance, including that of sphingolipid metabolism, glycerophospholipid metabolism, alanine, aspartate, and glutamate metabolism, and D-glutamine and D-glutamate metabolism. Our data may lead to a deeper understanding of the AD-associated metabolic profile and shed new light on the mechanism underlying the therapeutic effects of BSTSF.

## 1. Introduction

Alzheimer's disease (AD) is a common neurodegenerative disorder of the central nervous system characterized by progressive memory loss, cognitive impairment, abnormal behavior, and personality disorders. Dementia, including that related to AD, is the fifth leading cause of death worldwide, and 40–50 million people are thought to be affected by this condition [1], making it a major and increasing global health challenge. However, an effective treatment for AD remains elusive [2].

Traditional Chinese medicines (TCMs) have been used in the treatment of dementia for thousands of years. The Bushen Tiansui Formula (BSTSF, also known as “Naoling decoction”) is derived from Sagacious Confucius' Pillow Elixir, a classic Chinese medicinal formula mainly used to treat cognitive decline [3]. This formula comprises six herbs, including *Epimedium acuminatum* Franch. (Yinyanghuo), *Fallopia multiflora* (Thunb.) Harald. (Heshouwu), *Polygala tenuifolia* Willd. (Yuanzhi), *Acorus tatarinowii* Schott. (Shichangpu), *Plastrum testudinis* (Guiban), and *Ossa draconis* (Longgu). We recently showed that BSTSF could improve learning and memory deficits in AD model rats through regulating serum lipid metabolism and the amino acid metabolic pathway [4]. However, the mechanism underlying the therapeutic effects of BSTSF, especially its effects on metabolic stress and impaired lipid metabolism in brain tissue, is poorly understood.

Senile plaques, neurofibrillary tangles, and lipid granule accumulation were the three defining neuropathological features in the cerebral cortex of AD patients identified in the original analysis by Alois Alzheimer [5]. Subsequently, a large number of studies have shown that beta-amyloid (A*β*) plaques accumulate in the cerebral cortex in the early stages of AD [6–8]. These observations highlight that the cerebral cortex is the main pathological region in brain tissue of AD. The prefrontal cortex (PFC) is implicated in cognitive processes including working memory, temporal processing, decision-making, flexibility, and goal-oriented behavior [9]. Alterations in prefrontal cortex (PFC) function and abnormalities in its interactions with other brain areas (i.e., the hippocampus) have been related to Alzheimer's disease (AD) [10]. Several potential biomarkers have been proposed for preclinical AD and are mainly related to lipid metabolism. Perturbations of sphingolipid metabolism in brain tissue are consistently associated with endophenotypes across preclinical and prodromal AD, indicating that sphingolipids may be biologically relevant biomarkers for early AD detection [11]. Moreover, phosphatidylcholine breakdown may be mediated by phospholipase A2, leading to significantly elevated levels of glycerophosphocholine in human cerebrospinal fluid [12], while amino acids such as valine, arginine, and histidine are also associated with AD [13, 14]. These observations suggest that severe metabolic disorder and dysregulated lipid metabolism may have an important role in the pathogenesis of AD.

In this study, an ultra-high-performance liquid chromatography-mass spectrometry- (UHPLC-MS-) based metabolomic and lipidomic analysis was performed in cerebral cortices of control and AD model rats. Furthermore, the therapeutic effects of BSTSF and the mechanism underlying its ameliorating effects on the pathogenesis of AD were also explored for the first time using a metabolomic strategy. This study may provide a basis for a better understanding of the AD metabolic profile and novel insight into the clinical utility of BSTSF.

## 2. Material and Methods

### 2.1. Preparation of BSTSF

The six herbs (*H. epimedii*, *P. multiflorum*, tortoise plastron, *O. draconis*, *P. tenuifolia*, and R. acori graminei) comprising BSTSF were mixed at a ratio of 3 : 3 : 4 : 4 : 2 : 2. To ensure the quality of the herbal medicine, all herbs were obtained from the TCM pharmacy of the Second Xiangya Hospital of Central South University (CSU, Changsha, China) and were authoritatively identified by Professor Suiyu Hu. For details of the preparation and quality control of lyophilized powder, refer to our previous publication [15].

### 2.2. Animals and Experimental Design

Adult male Sprague-Dawley rats, weighing 180–220 g, used in our experiments, were purchased from the Laboratory Animal Centre of Central South University (Changsha, China). All animal experiments were performed following guidelines from the Committee on the Ethics of Animal Experiments of Central South University. Rats were housed under standard animal house conditions and randomly allocated into one of three groups: sham, AD, and BSTSF. For the AD and BSTSF groups, rats were injected intracerebroventricularly with oligomeric A*β*_1-42_ to generate a validated AD model, as we previously described [16]. The sham rats were injected bilaterally with the vehicle into the lateral ventricles. According to our previous study, the BSTSF group was intragastrically administrated with 27 g/kg BSTSF once daily from 1 to 28 days, whereas the sham and AD groups were intragastrically administrated with an equal volume of distilled water. By referring to the calculation formula from the *Experiment Methodology of Pharmacology*, the conversion factor between rats (200 g) and humans (70 kg) is 0.018; therefore, the calculated gavage dose of BSTSF for rats is about 9 g/kg/d. Our previous study explored the efficacy of three doses (9 g/kg/d, 27 g/kg/d, and 54 g/kg/d) of this prescription, and the result demonstrated that BSTSF owns optimal efficacy when it is administered at three times the regular dose; therefore, a dose of 27 g/kg/d was chosen for the experiments in the current study [15].

### 2.3. Morris Water Maze Test

The Morris water maze (MWM) test was used to assess the hippocampus-dependent learning and memory ability of the rats, as described in our previous study with minor modifications [17]. In brief, a spatial acquisition test was carried out from the 24th to 28th days after A*β*_1-42_ infusion, and animals were subjected to a five-day memory acquisition experiment to assess their spatial learning ability. Subsequent spatial probe experiments were conducted on day 29, to determine rat spatial memory retention ability. We applied SuperMaze video tracking and analysis systems to analyze experimental parameters (XR-XM101, Shanghai Softmaze Information Technology Co. Ltd., China).

### 2.4. UPLC-TripleTOF/MS-Based Metabolomics

#### 2.4.1. Sample Preparation

After the MWM test, the prefrontal cortices were harvested and immediately washed with precooled physiological saline and stored at −80°C for further analysis. Samples (50 mg) were accurately weighed, and the metabolites extracted using 400 *μ*L of a methanol : water (4 : 1, *v*/*v*) solution. The mixture was allowed to settle at −20°C and treated using a high-throughput Wonbio-96c tissue crusher (Shanghai Wanbo Biotechnology Co., Ltd., China) at 60 Hz for 6 min, vortexed for 30 s, and sonicated at 40 kHz for 10 min at −20°C. This step was performed three times. The samples were placed at −20°C for 30 min to precipitate the proteins. After centrifugation at 13,000 × *g* at 4°C for 15 min, the supernatants were carefully transferred to sample vials for LC-MS/MS analysis. The pooled quality control (QC) sample was prepared by mixing equal volumes of all the samples.

#### 2.4.2. Acquisition of LC-MS/MS Data

Metabolites were profiled using a UPLC-MS/MS-based platform. Chromatographic separation of the metabolites was performed on an ExionLC™ AD system (AB Sciex, USA) equipped with an ACQUITY UPLC BEH C18 column (100 mm × 2.1 mm i.d., 1.7 *μ*m) (Waters, Milford, CT, USA). The mobile phase consisted of 0.1% formic acid in water (solvent A) and 0.1% formic acid in acetonitrile : isopropanol (1 : 1, *v*/*v*) (solvent B). The solvent gradient program was as follows: from 0 to 3 min, 95% (A) : 5% (B) to 80% (A) : 20% (B); from 3 to 9 min, 80% (A) : 20% (B) to 5% (A) : 95% (B); from 9 to 13 min, 5% (A) : 95% (B) to 5% (A) : 95% (B); from 13 to 13.1 min, 5% (A) : 95% (B) to 95% (A) : 5% (B); and from 13.1 to 16 min, 95% (A) : 5% (B) to 95% (A) : 5% (B). The sample injection volume was 20 *μ*L, and the flow rate was set to 0.4 mL/min. The column temperature was maintained at 40°C. All these samples were stored at 4°C during the analysis. The UPLC system was coupled to a quadrupole time-of-flight mass spectrometer (TripleTOF™ 5600+; AB Sciex) equipped with an electrospray ionization (ESI) source operating in positive and negative modes. The optimal conditions were set as follows: source temperature, 500°C; curtain gas (CUR), 30 psi; ion sources GS1 and GS2, both 50 psi; ionspray voltage floating (ISVF), −4000 V in negative mode and 5000 V in positive mode; declustering potential, 80 V; and rolling collision energy (CE), 20–60 V for MS/MS analysis. Data acquisition was performed in the Data-Dependent Acquisition (DDA) mode. The detection was carried out over a mass range of 50–1000 *m*/*z*.

#### 2.4.3. Data Processing

After UPLC-MS/MS analysis, the raw data were imported into Progenesis QI 2.3 (Nonlinear Dynamics, Waters) for peak detection and alignment. The preprocessing results generated a data matrix that consisted of the retention time (RT), mass-to-charge ratio (*m*/*z*) values, and peak intensity. After filtering, half of the minimum metabolite values were imputed for specific samples in which the metabolite levels fell below the lower limit of quantitation and each metabolic feature was normalized by sum. The internal standard was used for data QC (reproducibility), and metabolic features with a QC relative standard deviation (RSD) > 30% were discarded. Following normalization and imputation, statistical analysis was performed on log-transformed data to identify significant differences in metabolite levels between comparable groups. The mass spectra of these metabolic features were identified by using the accurate mass, MS/MS fragment spectra, and isotope ratio difference searched in reliable metabolite databases such as the Human Metabolome Database (HMDB) (http://www.hmdb.ca/) and Metlin Database (https://metlin.scripps.edu/). Specifically, the mass tolerance between the measured *m*/*z* values and the exact mass of the components of interest was ±10 ppm. For metabolites confirmed by MS/MS, only those with a MS/MS fragment score above 30 were considered confidently identified. Otherwise, metabolites were only tentatively assigned.

#### 2.4.4. Statistical Analysis

Multivariate statistical analysis was performed using the ropls (version1.6.2) R package from Bioconductor on the Majorbio Cloud Platform (https://cloud.majorbio.com) and SIMCA-P 14.1 (Umetrics, Umea, Sweden). Unsupervised principal component analysis (PCA) was applied to obtain an overview of the metabolic data, general clustering, trends, and outliers. All the metabolite variables were scaled to unit variances before PCA. Orthogonal partial least squares discriminant analysis (OPLS-DA) was used to determine global metabolic changes between comparable groups. All the metabolite variables were scaled using Pareto scaling before the OPLS-DA. Model validity was evaluated from model parameters R2 and Q2, which provide information for the interpretability and predictability, respectively, of the model and avoid the risk of overfitting. Variable importance in the projection (VIP) was calculated in the OPLS-DA model. *p* values were estimated with paired Student's *t*-tests for single-dimensional statistical analysis. Significance among groups was assumed with VIP scores > 1 and *p* values < 0.05.

#### 2.4.5. Metabolic Pathway Analysis

Significantly altered metabolite data were imported into MetaboAnalyst 3.5 (https://www.metaboanalyst.ca) to investigate the therapeutic mechanisms related to BSTSF treatment. The impact value threshold calculated from pathway topology analysis was set to 0.10, and a raw *p* value < 0.05 was regarded as significant.

### 2.5. UHPLC-Obitrap/MS-Based Lipidomics

#### 2.5.1. Sample Preparation

Prefrontal cortex tissue (300 mg) was slowly thawed at 4°C and homogenized in 200 *μ*L of water. Then, 240 *μ*L of precooled methanol was added to the homogenate which was then vortexed for 10 s, mixed with 800 *μ*L of MTBE, vortexed again for 10 s, and finally sonicated for 20 min. The mixture was left at room temperature for 30 min and then centrifuged (14,000 × *g*, 10°C, 10 min). The upper layer was collected and dried using nitrogen. The samples were redissolved in 200 *μ*L of a 90% isopropanol/acetonitrile solution and then centrifuged (14,000 × *g*, 10°C, 10 min) before MS analysis. The supernatants were transferred into sample vials to be injected and analyzed by UHPLC-Obitrap/MS. The QC was prepared by mixing equal volumes of all the samples.

#### 2.5.2. Acquisition of LC-MS/MS Data

The UHPLC-Obitrap/MS analysis was performed in a UHPLC system (Nexera LC-30A, Shimadzu) and with a Q Exactive Plus mass spectrometer (Thermo Scientific). The UPLC autosampler temperature was set at 10°C, and the injection volume for each sample was 3 *μ*L. Column temperature was maintained at 45°C. The velocity was 300 *μ*L/min. The mobile phase consisted of acetonitrile and water (3 : 2) with 10 mM ammonium formate and 0.1% formic acid (solvent A) and isopropanol and acetonitrile (9 : 1) with 10 mM ammonium formate and 0.1% formic acid (solvent B). Mass spectrometry was performed in an either positive (ESI+) or negative (ESI−) electrospray ionization mode. The conditions for positive- and negative-ion modes were as follows: heater temperature, 300°C; sheath gas flow rate, 45 arb; auxiliary gas flow rate, 15 arb; sweep gas flow rate, 1 arb; spray voltage, 3.0 kV and 2.5 kV, respectively; capillary temperature, 350°C; S-lens RF level, 50% and 60%, respectively; and MS1 scan range: 200–1800 *m*/*z*. MS1 spectra were acquired with a target mass resolving power (RP) of 70,000 at *m*/*z* 200, and MS2 spectra were acquired with a target mass RP of 17,500 at *m*/*z* 200.

#### 2.5.3. Data Processing

LipidSearch software (version 4.1, Thermo Scientific) was used for peak identification, lipid identification (secondary identification), peak extraction, peak alignment, and quantitative processing. The main parameters were as follows: precursor tolerance, 5 ppm; product tolerance, 5 ppm; and product ion threshold, 5%. Lipid molecules with RSD > 30% were deleted.

### 2.6. Statistical Analysis

All data are presented as mean ± standard error of the mean (SEM) and were analyzed using SPSS 22.0 software (IBM Corp., Armonk, NY, USA). Student's *t*-test was carried out for comparisons between two groups, whereas ANOVA was conducted for comparisons of repeated measures. *p* < 0.05 was defined as indicating a statistically significant difference.

## 3. Results

### 3.1. BSTSF Ameliorates Learning and Memory Deficits of A*β*1-42-Induced AD Rats

**Figure 1:**
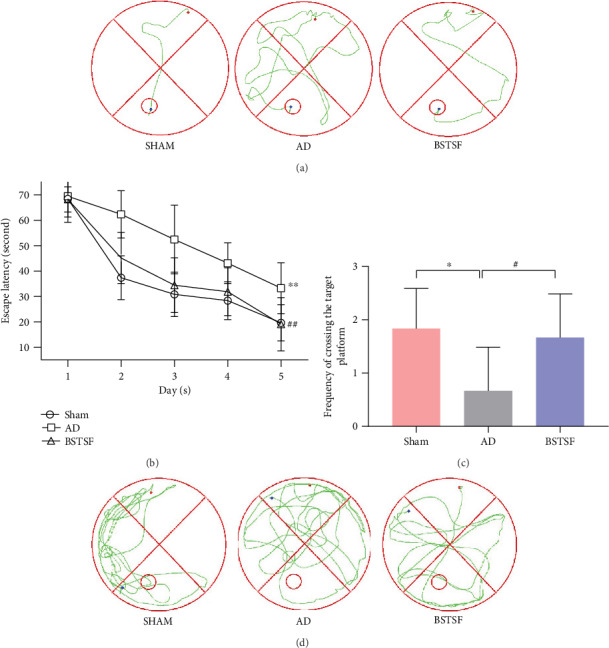
Effects of BSTSF on spatial learning and memory deficiency in AD rats. (a) Representative images of the swim paths and (b) time needed to reach the hidden platform. (c) Time spent in the target quadrant was measured for analysis of spatial memory function, and (d) representative images of the frequency of crossing the target platform within 90 seconds are shown. Data are expressed as the mean ± SD (*n* = 5 per group; escape latency was analyzed by repeated measures analysis of variance (ANOVA); other data were analyzed by one-way ANOVA followed by least significant difference tests). ^∗^*p* < 0.05, ^∗∗^*p* < 0.01 vs. sham group; ^#^*p* < 0.05, ^##^*p* < 0.05 vs. AD group.

First, we examined the effects of BSTSF on learning and memory ability in AD model rats using the MWM test. As shown in Figures 1(a) and 1(b), escape latency time gradually decreased in all groups over time; however, rats in the BSTSF group had significantly lower escape latency than those in the AD group during the last three training days. These results indicate that BSTSF can significantly alleviate the impaired learning ability induced by A*β*_1–42_. In the probe test, AD rats crossed the platform fewer times and spent less time in the target quadrant, suggesting that their memory capacity was significantly decreased, while BSTSF treatment significantly reversed these defects in AD rats (Figures 1(c) and 1(d)). Together, these results suggested that BSTSF treatment can ameliorate A*β*_1–42_-induced spatial learning and memory impairment.

### 3.2. Cerebral Cortex Metabolomics

#### 3.2.1. Metabolite Identification and Multivariate Statistical Analysis

**Figure 2:**
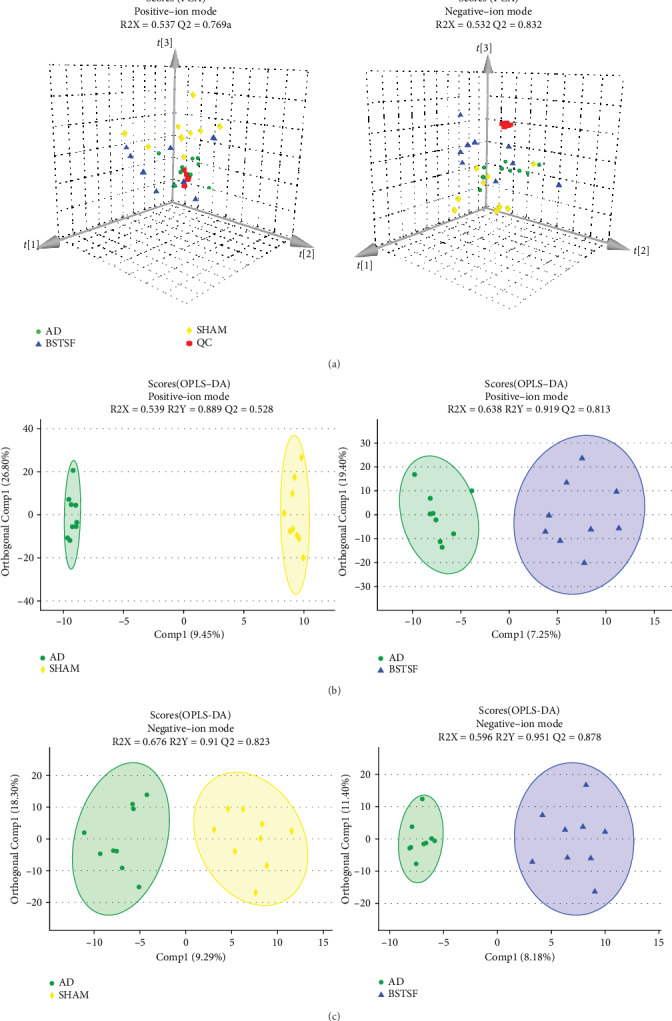
Multivariate statistical analysis of cerebral cortex metabolomics: (a) PCA 3D score plots of metabolomic data in the cerebral cortex and (b, c) OPLS-DA score plots between each two groups in positive- and negative-ion modes, respectively.

In this study, we identified 228 metabolites in a positive-ion mode and 287 in a negative-ion mode. An unsupervised PCA recognition model was generated for the whole dataset to evaluate the clustering trend of the samples with multidimensional data. The clustered QC samples confirmed the repeatability and stability of the instrument and the reliability of the data in the current research (Figure 2(a)). The separation between the AD and sham groups could be clearly observed in the PCA 3D patterns in both negative- and positive-ion modes, which demonstrated that the AD model can be successfully induced by A*β*1-42 and there was a severe metabolic disorder in the AD model rats. Furthermore, we also noted a trend towards separation between the BSTSF and AD groups, indicating that metabolism was significantly altered after 28 days of BSTSF administration. A supervised OPLS-DA pattern recognition method was applied to identify the overall metabolic differences between two groups. As shown in Figure 2(b) (positive-ion mode) and Figure 2(c) (negative-ion mode), a significant trend towards separation was observed between every two groups.

#### 3.2.2. Potential Metabolite Biomarkers in AD and Effect of BSTSF on AD

**Figure 3:**
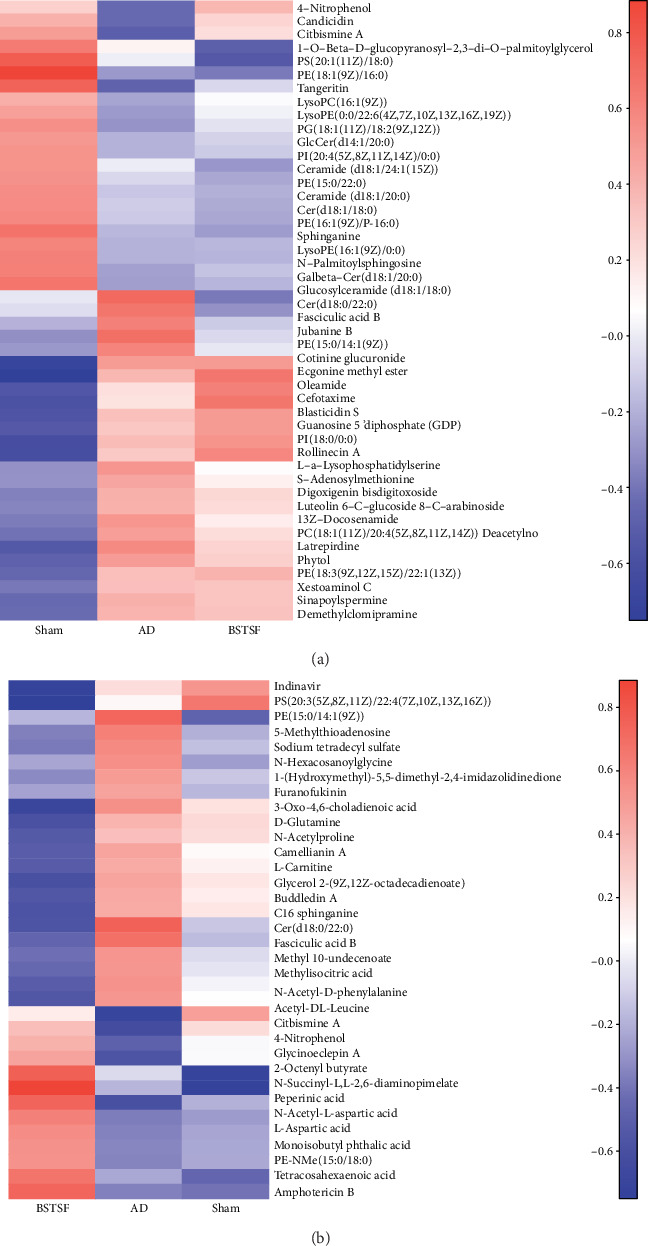
Heatmap of metabolites. (a) Heatmap analysis of the identified metabolites between groups sham and AD. (b) Heatmap analysis of the identified metabolites between groups BSTSF and AD. The blue band indicates a decreased level of metabolite, and the red band indicates an increased level of metabolite.

Metabolites with VIP scores > 1.0 and *p* values < 0.05 were defined as potential biomarkers. As shown in Table 1, forty-seven (24 upregulated and 23 downregulated) differential endogenous metabolites were identified between the AD and sham groups. After BSTSF administration, the levels of five metabolites changed significantly, including those of PE(15:0/14:1(9Z)), Cer(d18:0/22:0), fasciculic acid B, citbismine A, and 4-nitrophenol. 14 downregulated metabolites in the AD group compared with the sham group were increased after treatment with BSTSF, and 16 upregulated metabolites were decreased after administration with BSTSF. As shown in Table 2, thirty-four (12 upregulated and 22 downregulated) differential endogenous metabolites were identified between the BSTSF and AD groups. The differential metabolite dataset was imported into R (version 3.4.1) to generate a heatmap. Figures 3(a) and 3(b) show the 47 differential metabolites between the sham and AD groups and the 34 differential metabolites between the AD and BSTSF groups, as well as the relative changes in the concentration of the metabolites in the different groups.

#### 3.2.3. Analysis of Metabolic Pathways

**Figure 4:**
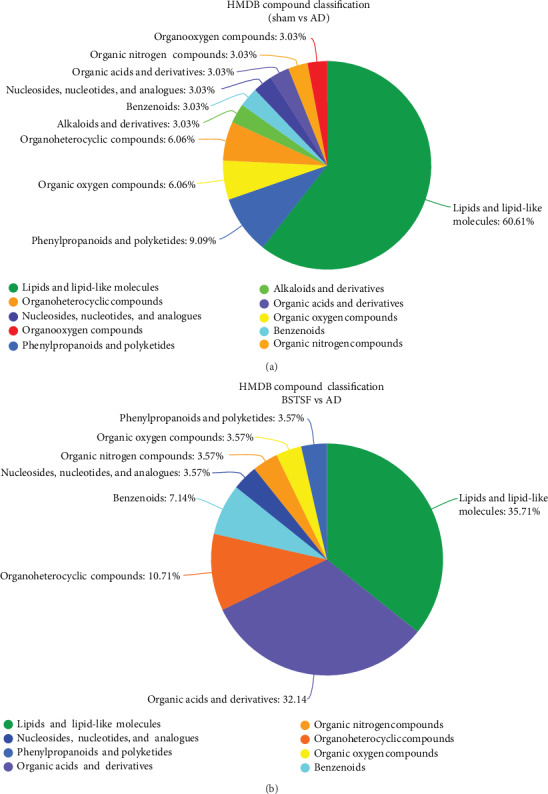
HMDB database classification: (a) differential metabolites between groups sham and AD in HMDB database classification and (b) differential metabolites between groups BSTSF and AD in HMDB database classification.

Metabolic pathway analysis was conducted with MetaboAnalyst 3.5 to further explore the pathogenesis of AD and the possible mechanism by which BSTSF treatment ameliorates this disease. The 47 differential endogenous metabolites between the sham and AD groups were mainly involved in sphingolipid metabolism, glycerophospholipid metabolism, linoleic acid metabolism, and alpha-linolenic acid metabolism (Table 3). The 34 differential endogenous metabolites between the BSTSF and AD groups were primarily associated with alanine, aspartate, and glutamate metabolism, D-glutamine and D-glutamate metabolism, arginine biosynthesis, and glycosylphosphatidylinositol (GPI) anchor biosynthesis (Table 4).  Figure 4 illustrates the differential metabolites classified through the HMDB database. The different colors in each pie chart represent different HMDB classifications, and the area represents the relative proportion of metabolites in the classification. The figure further shows that 60.61% of the differential metabolites between the AD and sham groups were lipid metabolites, indicative of significant changes in the levels of lipid metabolites in the cerebral cortices of AD rats. After BSTSF intervention, 35.71% of the differential metabolites were classified as significantly altered lipids.

### 3.3. Cerebral Cortex Lipidomics

#### 3.3.1. Lipid Identification and Multivariate Statistical Analysis

**Figure 5:**
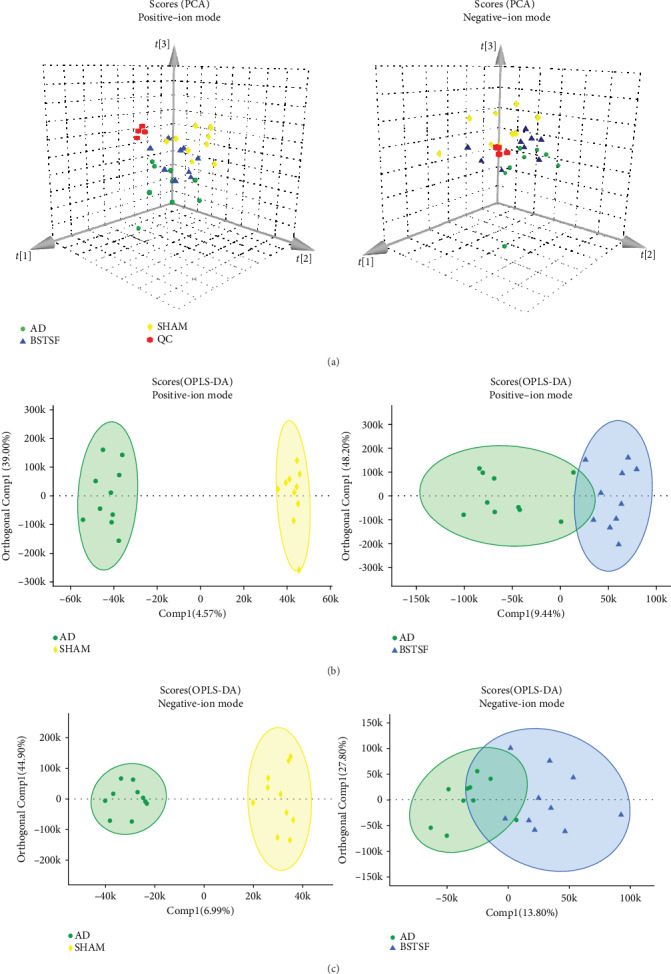
Multivariate statistical analysis of cerebral cortex lipidomics: (a) PCA 3D score plots of lipidomic data in the cerebral cortex and (b, c) OPLS-DA score plots between each two groups in positive- and negative-ion modes, respectively.

In this study, we identified 1191 lipid species from 31 lipid subclasses. The PCA 3D score plot chart (Figure 5(a)) shows a trend towards separation between the three groups. The clustered QC samples confirmed the repeatability and stability of the instrument and the reliability of the data. Moreover, OPLS-DA was performed to identify the pattern of separation. As shown in Figure 5(b) (positive-ion mode) and Figure 5(c) (negative-ion mode), there was a clear trend towards separation between the sham and AD groups and between the AD and BSTSF groups. This suggested that lipid metabolism was dysregulated in the cerebral cortices of AD rats, and this dysregulation could be ameliorated by BSTSF administration.

#### 3.3.2. Identification of the Differential Lipid Metabolites in Cerebral Cortex Samples between the Sham and AD Groups and the BSTSF and AD Groups

**Figure 6:**
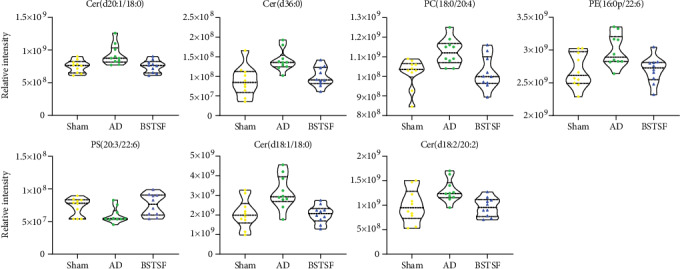
Changes in the relative signal intensities of differential lipids in the rat cerebral cortex from different groups.

VIP scores > 1 and *p* values < 0.05 were used to determine the significantly altered lipid metabolites between the sham and AD groups and the BSTSF and AD groups. As shown in Table 5, the concentrations of 30 lipids changed markedly after being induced by A*β*1-42 while those of 21 lipids were altered after BSTSF administration. Interestingly, ceramide levels, including those of Cer(d18:1/18:0), Cer(d18:2/20:2), Cer(d36:0), and Cer(d20:1/18:0), and PC(18:0/20:4) and PE(16:0p/22:6) were substantially higher in the AD group than in the sham group, whereas the levels of all these metabolites were significantly reduced in BSTSF-treated rats. PS(20:3/22:6) was downregulated in the AD group compared with the sham group; however, its level was markedly upregulated with BSTSF treatment (Figure 6).

## 4. Discussion

**Figure 7:**
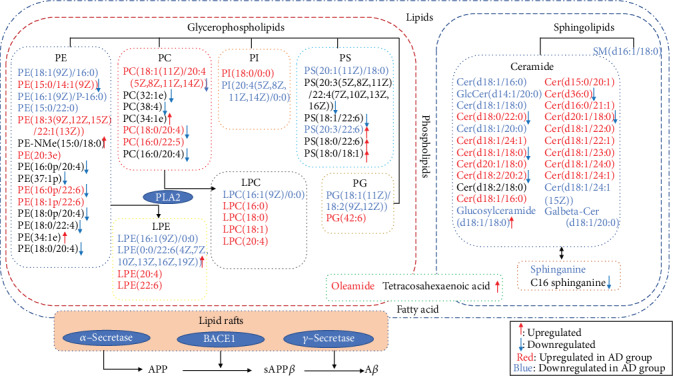
Lipid metabolism pathway map depicts the pathomechanism in AD rats and the therapeutic mechanism of BSTSF for AD. Notes: the red and blue letters indicate that the lipids were upregulated or downregulated, respectively, in group AD compared with group sham. The red upward arrow and blue downward arrow indicate that the lipids were upregulated or downregulated, respectively, by BSTSF.

In the present study, we found that the cerebral cortex of the AD rats has a distinct metabolomic profile, including the dysregulated sphingolipid metabolism, glycerophospholipid metabolism, linoleic acid metabolism, and alpha-linolenic acid metabolism. The lipidomic analysis indicated that sphingolipids and glycerophospholipids, such as Cer (ceramide), PE (phosphatidylethanolamine), LPE (lysophosphatidylethanolamine), and PC (phosphatidylcholine), are dysregulated in the brain of AD rats. Moreover, the results indicated that BSTSF treatment could restore some dysregulation metabolites and abnormal lipid metabolism in the cerebral cortex of AD rats, especially involving lipid and amino acid metabolism (Figure 7).

Lipid homeostasis plays important roles in the central nervous system, including the maintenance of cell membrane structure, signal transduction, and being components of lipid rafts [18–20]. Our study identified sphingolipids as a class of lipid metabolites that are closely related to the pathology of AD. As the key intermediates in sphingolipid metabolism, we found that the levels of Cer(d18:0/22:0, d18:1/18:0, d18:2/20:2, d36:0, d20:1/18:0) were markedly upregulated in the AD group when compared with the sham-treated group. Some studies demonstrated that elevated Cer levels in reactive astrocytes were associated with neuroinflammation [21] and can promote the overproduction and aggregation of A*β* through their effects on lipid rafts [22]. Interestingly, the Cer levels were significantly reduced after BSTSF administration and may act as a putative therapeutic target of BSTSF in AD. In addition, sphingomyelins (SMs) were a subclass of sphingolipids, and it also enriched in the central nervous system. They are important constituents of lipid rafts [23] and play a critical role in neuronal cell signaling [24]. Importantly, SMs have been associated with amyloidogenic processing of the amyloid precursor protein (APP) [25]. Here, we showed that the levels of SM(d16:1/18:0) were downregulated in AD brains when compared with those from the sham-treated group.

Glycerophospholipids were the second major class of lipid metabolites found to be related to AD pathology and BSTSF therapeutic mechanisms in the present study. Concentrations of glycerophospholipids have been associated with the severity of amyloid and neurofibrillary pathology in AD [26]. In this study, the levels of PE(15:0/14:1(9Z)), PE(16:0p/22:6), LPE(0:0/22:6(4Z,7Z,10Z,13Z,16Z,19Z)), PC(18:1(11Z)/20:4(5Z,8Z,11Z,14Z)), PC(18:0/20:4), and PS(20:3/22:6) were dysregulated in AD rats when compared with those of rats in the sham group; however, this dysregulation was ameliorated after BSTSF treatment. A related study showed that BACE1 silencing restored PE derivatives such as LPE and etherphosphatidylethanolamine and reduced PLA2 phosphorylation, which favored cellular homeostasis and cognitive function recovery in the hippocampus of triple transgenic AD mice [27]. Similar to our findings, the level of PC was increased in cerebrospinal fluid from AD patients and was associated with aberrant cerebrospinal fluid A*β*1−42 values, which may be indicative of loss of membrane function and neurodegeneration in the early stages of cognitive dysfunction [14]. Our data confirmed that the concentrations of PC(18:1(11Z)/20:4(5Z,8Z,11Z,14Z)) and PC(18:0/20:4) were increased in the cerebral cortex of the AD rat, which may facilitate the aggregation of A*β*. Treatment with BSTSF may reduce A*β* aggregation through modulation of related PCs. Myelin is enriched in PS in brain tissue, and the docosahexaenoic acid (DHA) content of neuronal PS is of functional importance. Lower PS concentration can reduce the sensitivity of postsynaptic membranes to neurotransmitters such as acetylcholine [28]. PE was also reported to reduce reactive oxygen species levels and protect neuron membranes from oxidative damage [29]. One study found that PS supplementation significantly improved memory, information processing, and the ability to perform daily activities in elderly people [30]. Our data showed that the concentration of PS(20:3/22:6) was markedly upregulated in BSTSF-treated AD rats, which might ameliorate AD pathology by improving sensitivity to neurotransmitters and reducing oxidative damage.

The metabolites of sphingolipids and glycerophospholipids both belonged to phospholipid metabolism. So there was a serious disturbance of phospholipid metabolism in brain tissue of AD. Related research confirmed that ApoE proteins are critical determinants of brain phospholipid homeostasis. The phospholipid dysregulation contributes to ApoE4-associated cognitive deficits in AD pathogenesis [31]. APOE4 has been identified as the most prevalent genetic risk factor for AD [32], and it could exacerbate the intraneuronal accumulation of A*β* [33] and plaque deposition in the brain parenchyma [34]. Therefore, BSTSF might treat AD through regulating the APOE4 and phospholipid metabolism.

Additionally, we observed that the level of oleamide was upregulated in AD rats while that of tetracosahexaenoic acid was downregulated after BSTSF administration, and both of these lipids have roles in fatty acid metabolism. Oleamide has been found to accumulate in the cerebrospinal fluid of sleep-deprived cats and mice [35] and is an important regulatory lipid in the brain and central nervous system. Oleamide regulates the sleep-wake cycle, memory, locomotion, and pain perception and exhibits anti-inflammatory, anxiolytic, and neuroprotective properties [36]. Oleamide levels were shown to be increased in the plasma of AD patients [37]. A recent study indicated that DHA is both a product and a precursor of tetracosahexaenoic acid [38], while other investigations have shown that dietary DHA administration can exert protective effects against A*β* production, plaque deposition, and cerebral amyloid angiopathy in an aged mouse model of AD, as well as increasing cerebral blood volume [39, 40]. This indicates that BSTSF might regulate DHA and increase tetracosahexaenoic acid treating AD.

In the current study, the level of S-adenosylmethionine (SAM) was significantly upregulated in the cerebral cortex of the AD rat, indicative of aberrant amino acid metabolism in AD. S-Adenosylmethionine is generated through the activity of methionine adenosyltransferases, and more than 50 endogenous substances in the body, including phosphatidylcholine, need SAM as the methyl donor. Homocysteine is formed by SAM demethylation. A related study showed that an elevated total homocysteine baseline serum level during six years was associated with an increased risk of dementia and AD, and the putative mechanism was associated with loss of total brain tissue volume and brain atrophy [41, 42]. Therefore, increased SAM concentrations in the AD brain may lead to higher levels of homocysteine, resulting in the loss of total brain tissue volume and brain atrophy. We found that BSTSF ameliorated AD pathology mainly through the alanine, aspartate, and glutamate metabolic pathway and the D-glutamine and D-glutamate metabolic pathway that is involved in regulating amino acid metabolism. N-Acetyl-L-aspartic acid (NAA) and L-aspartic acid are involved in the alanine, aspartate, and glutamate metabolic pathway. NAA is a biomarker for neuronal damage in the human brain during neurodegeneration [43], and reduced cortical levels of NAA were shown to be correlated with clinical scales of dementia severity [44]. L-Aspartic acid also functions as a neurotransmitter. D-Glutamine forms part of the D-glutamate metabolic pathway and is converted from glutamate by astrocytes in the brain [45]. Glutamate, as an excitatory neurotransmitter, is released decarboxylated by neurons to yield the inhibitory neurotransmitter gamma-aminobutyric acid (GABA) [46]. Numerous studies have shown that the level of GABA is closely associated with AD [47, 48]. D-Glutamine and GABA could be putative therapeutic targets of BSTSF in AD.

Through integrated metabolic and lipidomic analyses, we revealed the metabolic dysregulation in the cerebral cortex of the AD rat, as well as the metabolism-related therapeutic effects of BSTSF on AD. However, this study had some limitations. First, we only obtained a relative quantification of metabolite levels based on untargeted metabolomic analysis. Absolute quantitation of critical metabolites performed by targeted metabolomics is needed in future research. Second, numerous metabolites are regulated by BSTSF; however, the associated molecular mechanisms are not well understood and will be the basis of our future research.

## 5. Conclusions

The metabolic disturbance in the cerebral cortex of the AD rat is primarily associated with sphingolipid metabolism, glycerophospholipid metabolism, linoleic acid metabolism, and fatty acid metabolism. Moreover, BSTSF ameliorates the severity of AD by regulating phospholipid metabolism, maintaining fatty acid metabolism, and balancing amino acid metabolism. Our research highlighted some important mechanisms involved in the pathogenesis of AD and revealed the metabolism-related therapeutic effects of BSTSF on AD.

## Figures and Tables

**Table 1:** Differentially expressed endogenous metabolites between groups sham and AD and their change trends in all groups.

Metabolite	*m*/*z*	Rt (min)	HMDB ID	PubChem ID	AD vs. sham			AD^a^	BSTSF^b^
					VIP	*p*	FC		
ESI+									
Tangeritin	373.1267	5.3139	0030539	68077	2.374	0.001	0.591	↓^∗^	↑
Cer(d18:1/16:0)	538.5199	11.3614	0000790	5283564	1.953	0.004	0.750	↓^∗^	↑
Candicidin	566.289	7.6092	0015283	10079874	1.493	0.031	0.835	↓^∗^	↑
Cer(d18:1/24:1(15Z))	648.6289	13.1778	0004953	5283568	1.468	0.035	0.871	↓^∗^	—
GlcCer(d14:1/20:0)	700.5725	11.005	—	70699223	1.257	0.043	0.883	↓^∗^	↑
Galbeta-Cer(d18:1/20:0)	778.6169	12.1307	—	44260150	1.564	0.012	0.902	↓^∗^	↑
Cer(d18:1/18:0)	566.5511	11.9439	0004950	5283565	2.03	0.004	0.929	↓^∗^	—
Sphinganine	302.3053	7.4398	0000269	91486	1.656	0.014	0.949	↓^∗^	—
LysoPE(16:1(9Z)/0:0)	452.2772	7.6754	0011504	52925129	1.644	0.021	0.976	↓^∗^	↑
PE(18:1(9Z)/16:0)	759.565	8.4109	0009055	9546802	2.422	0.001	0.977	↓^∗^	—
LysoPC(16:1(9Z)/0:0)	494.3241	7.5904	0010383	24779461	1.4	0.041	0.986	↓^∗^	↑
PI(20:4(5Z,8Z,11Z,14Z)/0:0)	621.303	7.8276	—	42607497	1.679	0.023	0.986	↓^∗^	↑
LysoPE(0:0/22:6(4Z,7Z,10Z,13Z,16Z,19Z))	548.2745	7.6944	0011496	53480945	1.603	0.043	0.989	↓^∗^	↑
Oleamide	563.5505	8.9533	0002117	5283387	2.205	0.001	1.003	↑^∗^	—
Cer(d18:0/22:0)	665.6552	10.5332	0011765	5283575	1.564	0.01	1.004	↑^∗^	↓^∗^
Desmethylclomipramine	301.1428	13.9123	0060947	622606	1.551	0.026	1.005	↑^∗^	↓
Phytol	360.3263	13.3117	0002019	5280435	1.649	0.017	1.007	↑^∗^	↓
PE(15:0/14:1(9Z))	680.4797	10.8995	0008888	52924158	1.879	0.014	1.017	↑^∗^	↓^∗^
Cotinine glucuronide	416.1429	0.768	0001013	3398121	1.78	0.025	1.024	↑^∗^	↓
13Z-Docosenamide	338.3424	9.7988	—	5365371	1.091	0.05	1.027	↑^∗^	↓
PI(18:0/0:0)	601.3349	9.4901	—	42607495	1.735	0.011	1.027	↑^∗^	—
L-a-Lysophosphatidylserine	526.3142	9.1446	—	28040605	1.827	0.002	1.029	↑^∗^	—
S-Adenosylmethionine	399.1444	0.5786	0001185	34755	1.358	0.033	1.038	↑^∗^	↓
Rollinecin A	663.4538	11.419	0030438	177320	1.881	0.005	1.046	↑^∗^	—
Jubanine B	762.3905	6.2048	0030206	101316795	1.692	0.011	1.052	↑^∗^	↓
Sinapoylspermine	409.2812	6.1949	0033479	131751433	1.891	0.005	1.052	↑^∗^	↓
Latrepirdine	352.2404	12.5435	0240240	197033	1.828	0.002	1.059	↑^∗^	↓
Ecgonine methyl ester	232.1546	12.0323	0006406	104904	2.296	0.001	1.085	↑^∗^	—
Xestoaminol C	230.248	6.1084	—	14756407	1.128	0.044	1.087	↑^∗^	↓
Fasciculic acid B	678.4588	7.1307	0036438	196808	1.434	0.011	1.140	↑^∗^	↓^∗^
ESI-									
Citbismine A	639.1921	3.8775	0041086	131753020	1.875	0.007	0.840	↓^∗^	↑^∗^
Cer(d18:1/20:0)	638.5713	12.6161	0004951	5283566	2.051	0.004	0.916	↓^∗^	↑
Glucosylceramide (d18:1/18:0)	772.5938	11.5561	0004972	11958364	1.543	0.009	0.929	↓^∗^	↑
1-O-Beta-D-glucopyranosyl-2,3-di-O-palmitoylglycerol	775.5571	11.6171	0031680	10462651	1.902	0.005	0.941	↓^∗^	—
PE(16:1(9Z)/P-16:0)	672.4954	11.2227	0008982	53479605	1.331	0.038	0.954	↓^∗^	—
PC(18:1(11Z)/18:2(9Z,12Z))	771.5158	10.4438	0010620	53480619	1.676	0.027	0.962	↓^∗^	↑
PE(15:0/22:0)	806.5909	11.8787	0008907	52924172	1.69	0.025	0.974	↓^∗^	—
PS(20:1(11Z)/18:0)	838.5596	12.6161	0112545	52925649	2.134	0.006	0.977	↓^∗^	—
4-Nitrophenol	138.02	3.6423	0001232	980	1.926	0.015	1.014	↓^∗^	↑^∗^
PC(18:1(11Z)/20:4(5Z,8Z,11Z,14Z))	852.5756	10.6464	0008081	53478741	1.665	0.019	1.019	↑^∗^	↓
PE(18:3(9Z,12Z,15Z)/22:1(13Z))	840.5778	10.9036	0009172	53479688	1.712	0.01	1.022	↑^∗^	
GDP	442.0154	0.883	0001201	135398619	1.617	0.006	1.042	↑^∗^	—
Luteolin 6-C-glucoside 8-C-arabinoside	655.1563	11.4852	0029258	131750830	1.708	0.046	1.067	↑^∗^	↓
Digoxigenin bisdigitoxoside	631.3473	10.9633	0060818	92999	1.397	0.045	1.155	↑^∗^	↓
Blasticidin S	459.1466	4.0897	0030452	170012	1.823	0.016	1.221	↑^∗^	—
Cefotaxime	490.0302	4.0897	0014636	5742673	1.656	0.043	1.715	↑^∗^	—
Deacetylnomilin	509.1563	3.6723	0035684	90472146	1.548	0.018	0.591	↓^∗^	—

Abbreviations: AD: Alzheimer's disease; Rt (min): retention time; VIP: variable importance; FC: fold change. Fold change was calculated as relative intensity obtained from group sham/group AD, and a value less than 1 indicates a decrease in the metabolites of group AD. The levels of potential biomarkers were labeled with “↓” (downregulated) and “↑” (upregulated) (^∗^*p* < 0.05). ^a^Change trend compared with the sham group. ^b^Change trend compared with the AD group.

**Table 2:** Differentially expressed endogenous metabolites between groups BSTSF and AD and their change trends in all groups.

Metabolite	*m*/*z*	Rt (min)	HMDB ID	PubChem ID	BSTSF vs. AD			AD^a^	BSTSF^b^
					VIP	*p*	FC		
ESI+									
5′-Methylthioadenosine	342.0866	2.1028	0001173	439176	1.779	0.028	0.962	↑	↓^∗^
1-(Hydroxymethyl)-5,5-dimethyl-2,4-imidazolidinedione	123.0551	1.3183	0031670	67000	1.923	0.031	0.981	↑	↓^∗^
Buddledin A	277.1805	6.8324	—	5281514	2.010	0.022	0.953	↑	↓^∗^
C16 sphinganine	274.2742	5.8082	—	5283572	2.003	0.029	0.981	↑	↓^∗^
D-Glutamine	147.0762	0.7581	0003423	145815	1.907	0.032	0.987	↑	↓^∗^
Cer(d18:0/22:0)	665.6552	10.5332	0011765	5283575	2.332	0.007	0.995	↑^∗^	↓^∗^
Fasciculic acid B	678.4588	7.1307	0036438	196808	1.923	0.021	0.833	↑^∗^	↓^∗^
L-Carnitine	162.1123	0.7298	0000062	10917	2.276	0.005	0.981	↑	↓^∗^
Methyl 10-undecenoate	199.1691	6.5087	0029585	8138	1.762	0.038	0.992	↑	↓^∗^
PE(15:0/14:1(9Z))	680.4797	10.8995	0008888	52924158	2.101	0.017	0.987	↑^∗^	↓^∗^
N-Acetylproline	190.1068	5.3884	0094701	66141	1.920	0.028	0.987	↑	↓^∗^
N-Succinyl-L,L-2,6-diaminopimelate	273.1097	14.0721	0012267	25202447	1.759	0.038	1.007		↑^∗^
Peperinic acid	205.0849	6.1559	0038181	156203	2.192	0.008	1.013	—	↑^∗^
2-Octenyl butyrate	199.1693	7.5996	0038081	124355627	1.989	0.019	1.045	↓	↑^∗^
Glycinoeclepin A	510.2488	5.5944	0037037	19007174	1.914	0.022	1.020	↓	↑^∗^
L-Aspartic acid	134.0446	0.9605	0000191	5960	1.741	0.040	1.008	↓	↑^∗^
Monoisobutyl phthalic acid	205.085	5.9735	0002056	92272	2.182	0.008	1.028	↓	↑^∗^
N-Acetyl-L-aspartic acid	351.1035	0.9605	0000812	65065	2.425	0.003	1.018	↓	↑^∗^
ESI-									
3-Oxo-4,6-choladienoic acid	415.2505	14.0629	0000476	5283992	2.242	0.007	0.981	↑	↓^∗^
Acetyl-DL-leucine	172.0975	2.7628	—	1995	2.152	0.012	0.882	↑	↓^∗^
Camellianin A	655.1471	10.0921	0029908	5487343	2.046	0.020	0.985	↑	↓^∗^
Furanofukinin	293.1751	5.9909	0036640	78385403	1.662	0.042	0.981	↑	↓^∗^
Glycerol 2-(9Z,12Z-octadecadienoate)			0041511	15607291	1.992	0.025	0.871	—	↓^∗^
Indinavir	648.3376	11.0497	0014369	5362440	1.912	0.029	0.972		↓^∗^
Methylisocitric acid	241.0113	0.73	0006471	5459784	1.897	0.036	0.939	↑	↓^∗^
N-Acetyl-D-phenylalanine	206.0817	3.0047	—	101184	1.863	0.027	0.887	↑	↓^∗^
N-Hexacosanoylglycine	498.4148	9.9473	0062678	91828268	1.704	0.046	0.798	↑	↓^∗^
PS(20:3(5Z,8Z,11Z)/22:4(7Z,10Z,13Z,16Z))	896.518	12.6161	0112616	131819845	1.904	0.031	0.955	—	↓^∗^
Sodium tetradecyl sulfate	275.1677	7.6509	0014607	23665770	1.752	0.032	0.841	↑	↓^∗^
Tetracosahexaenoic acid	393.2201	6.0111	0002007	6439582	1.912	0.033	1.290	↓	↑^∗^
4-Nitrophenol	138.02	3.6423	0001232	980	1.675	0.034	1.028	↓^∗^	↑^∗^
Amphotericin B	922.4847	10.5551	0014819	5280965	2.121	0.016	1.102		↑^∗^
Citbismine A	639.1921	3.8775	0041086	131753020	1.609	0.038	1.710	↓^∗^	↑^∗^
PE-NMe(15:0/18:0)	754.5181	11.0497	0113019	131820134	1.896	0.021	1.051	↓	↑^∗^

Abbreviations: BSTSF: Bushen Tiansui Formula; AD: Alzheimer's disease; Rt (min): retention time; VIP: variable importance; FC: fold change. Fold change was calculated as average relative quantitation obtained from group BSTSF/group AD, and a value less than 1 indicates a decrease in the metabolites of group BSTSF. The levels of potential biomarkers were labeled with “↓” (downregulated) and “↑” (upregulated) (^∗^*p* < 0.05). ^a^Change trend compared with the sham group. ^b^Change trend compared with the AD group.

**Table 3:** Metabolite pathway changes between sham and AD groups.

No.	Pathway	Hits	Total	*p*	−Log(*p*)	FDR *p*	Impact
1	Sphingolipid metabolism	3	21	0.0011		0.0901	0.46248
2	Glycerophospholipid metabolism	3	36	0.0052	5.2518	0.21999	0.21631
3	Linoleic acid metabolism	1	5	0.0506	2.9834	1	0
4	Alpha-linolenic acid metabolism	1	13	0.1266	2.0663	1	0
5	Glycosylphosphatidylinositol (GPI) anchor biosynthesis	1	14	0.1357	1.997	1	0.00399
6	Cysteine and methionine metabolism	1	33	0.2925	1.2293	1	0.05271
7	Arachidonic acid metabolism	1	36	0.3147	1.1562	1	0
8	Arginine and proline metabolism	1	38	0.3291	1.1115	1	0
9	Purine metabolism	1	65	0.4979	0.69746	1	0.02939

Hits represent the matched number of metabolites in one pathway. *p* represents the original *p* value calculated from the enrichment analysis. FDR *p* represents the *p* value adjusted using the false discovery rate.

**Table 4:** Metabolite pathway changes between BSTSF and AD groups.

No.	Pathway	Hits	Total	*p*	−Log(*p*)	FDR *p*	Impact
1	Alanine, aspartate, and glutamate metabolism	2	28	0.0156	4.157	1	0.3101
2	D-Glutamine and D-glutamate metabolism	1	6	0.0419	3.1725	1	0
3	Arginine biosynthesis	1	14	0.0953	2.3509	1	0
4	Glycosylphosphatidylinositol (GPI) anchor biosynthesis	1	14	0.0953	2.3509	1	0.00399
5	Nicotinate and nicotinamide metabolism	1	15	0.1017	2.2851	1	0
6	Histidine metabolism	1	16	0.1082	2.2238	1	0
7	Pantothenate and CoA biosynthesis	1	19	0.1273	2.0615	1	0
8	Beta-alanine metabolism	1	21	0.1398	1.9678	1	0
9	Cysteine and methionine metabolism	1	33	0.2114	1.554	1	0.02089
10	Glycerophospholipid metabolism	1	36	0.2284	1.4765	1	0.10449
11	Aminoacyl-tRNA biosynthesis	1	48	0.2933	1.2265	1	0

Notes: hits represent the matched number of metabolites in one pathway. *p* represents the original *p* value calculated from the enrichment analysis. FDR *p* represents the *p* value adjusted using the false discovery rate.

**Table 5:** Differentially expressed lipids detected by LC-MS/MS.

Lipid ion	Class	Ion formula	*m*/*z*	Rt (min)	AD vs. sham			BSTSF vs. AD		
					*p*	VIP	FC	*p*	VIP	FC
ESI+										
Cer(d18:1/24:1)	Cer	C42 H82 O3 N1	648.6289	18.24252	0.016	1.420	1.373	—	—	—
PE(20:3e)	PE	C25 H46 O7 N1 P1 Na1	526.2904	3.140912	0.004	1.102	1.646	—	—	—
Cer(d18:1/18:0)	Cer	C36 H72 O3 N1	566.5507	15.29891	0.007	6.805	1.529	0.002	1.724	0.646
Cer(d20:1/18:0)	Cer	C38 H76 O3 N1	594.582	16.73516	0.003	1.898	1.507	—	—	—
LPC(16:0)	LPC	C24 H51 O7 N1 P1	496.3398	4.266847	0.041	2.016	1.533	—	—	—
Cer(d18:2/20:2)	Cer	C38 H70 O3 N1	588.535	15.19771	0.033	3.174	1.304	0.003	2.537	0.747
LPC(18:0)	LPC	C26 H55 O7 N1 P1	524.3711	5.993806	0.046	1.211	1.417	—	—	—
PC(32:1e)	PC	C40 H81 O7 N1 P1	718.5745	14.22706	—	—	—	0.007	1.014	0.718
PC(38:4)	PC	C46 H85 O8 N1 P1	810.6007	13.76129	—	—	—	0.011	3.016	0.872
PE(16:0p/20:4)	PE	C41 H75 O7 N1 P1	724.5276	13.45675	—	—	—	0.018	2.391	0.778
Cer(d18:2/18:0)	Cer	C36 H70 O3 N1	564.535	14.0985	—	—	—	0.021	1.147	0.784
PE(37:1p)	PE	C42 H82 O7 N1 P1 Na1	766.5721	13.06318	—	—	—	0.030	1.838	0.735
PS(18:1/22:6)	PS	C46 H77 O10 N1 P1	834.528	11.2969	—	—	—	0.033	1.718	0.663
PC(34:1e)	PC	C42 H85 O7 N1 P1	746.6058	15.56148	—	—	—	0.042	2.541	1.515
ESI-										
PS(20:3/22:6)	PS	C48 H75 O10 N1 P1	856.5134	11.75541	0.030	1.406	0.815	0.021	1.537	1.279
SM(d16:1/18:0)	SM	C40 H80 O8 N2 P1	747.5658	11.40332	0.020	1.280	0.817	—	—	—
Cer(d18:1/16:0)	Cer	C35 H68 O5 N1	582.5103	12.79807	0.009	1.942	1.367	—	—	—
Cer(d15:0/20:1)	Cer	C36 H70 O5 N1	596.5259	13.38667	0.004	1.052	1.439	—	—	—
Cer(d36:0)	Cer	C37 H74 O5 N1	612.5572	14.35006	0.002	2.378	1.598	0.002	1.160	0.704
Cer(d16:0/21:1)	Cer	C38 H74 O5 N1	624.5572	14.49407	0.014	1.254	1.464	—	—	—
Cer(d20:1/18:0)	Cer	C39 H76 O5 N1	638.5729	15.25846	0.002	4.956	1.382	0.008	2.903	0.811
Cer(d18:1/22:0)	Cer	C41 H80 O5 N1	666.6042	16.5813	0.000	1.921	1.404	—	—	—
Cer(d18:1/22:1)	Cer	C41 H78 O5 N1	664.5885	15.15109	0.018	1.171	1.486	—	—	—
Cer(d18:1/23:0)	Cer	C42 H82 O5 N1	680.6198	17.28717	0.001	1.368	1.431	—	—	—
Cer(d18:1/24:0)	Cer	C43 H84 O5 N1	694.6355	17.99214	0.002	1.893	1.423	—	—	—
Cer(d18:1/24:1)	Cer	C43 H82 O5 N1	692.6198	16.51347	0.014	3.881	1.372	—	—	—
LPC(16:0)	LPC	C25 H51 O9 N1 P1	540.3307	3.236844	0.000	1.157	1.460	—	—	—
LPC(18:0)	LPC	C27 H55 O9 N1 P1	568.362	4.929942	0.016	1.612	1.386	—	—	—
LPC(18:1)	LPC	C27 H53 O9 N1 P1	566.3463	3.43383	0.002	1.347	1.474	—	—	—
LPC(20:4)	LPC	C29 H51 O9 N1 P1	588.3307	2.702941	0.023	1.167	1.610	—	—	—
LPE(20:4)	LPE	C25 H43 O7 N1 P1	500.2783	2.830786	0.002	1.657	1.552	—	—	—
LPE(22:6)	LPE	C27 H43 O7 N1 P1	524.2783	2.696826	0.002	2.536	1.640	—	—	—
PC(18:0/20:4)	PC	C47 H85 O10 N1 P1	854.5917	12.64714	0.004	1.505	1.103	0.007	1.605	0.907
PC(16:0/22:5)	PC	C47 H83 O10 N1 P1	852.576	12.042	0.046	1.805	1.422	—	—	—
PE(16:0p/22:6)	PE	C43 H73 O7 N1 P1	746.513	11.99096	0.016	3.248	1.112	0.009	3.056	0.902
PE(18:1p/22:6)	PE	C45 H75 O7 N1 P1	772.5287	12.0979	0.009	1.202	1.104	—	—	—
PG(42:6)	PG	C48 H82 O10 N0 P1	849.5651	11.75907	0.022	1.445	1.248	—	—	—
PC(16:0/20:4)	PC	C45 H81 O10 N1 P1	826.5604	11.53414	—	—	—	0.005	1.825	0.887
PE(18:0p/20:4)	PE	C43 H77 O7 N1 P1	750.5443	13.11735	—	—	—	0.009	2.226	0.894
PE(18:0/22:4)	PE	C45 H81 O8 N1 P1	794.5705	13.64911	—	—	—	0.017	1.971	0.905
PS(18:0/22:6)	PS	C46 H77 O10 N1 P1	834.5291	13.47165	—	—	—	0.027	1.376	2.309
PE(34:1e)	PE	C39 H77 O7 N1 P1	702.5443	13.497	—	—	—	0.030	1.789	1.250
PE(18:0/20:4)	PE	C43 H77 O8 N1 P1	766.5392	12.85352	—	—	—	0.038	2.780	0.924
PS(18:0/18:1)	PS	C42 H79 O10 N1 P1	788.5447	12.64461	—	—	—	0.044	2.484	1.284

Notes: BSTSF: Bushen Tiansui Formula; AD: Alzheimer's disease; RT (min): retention time; VIP: variable importance; FC: fold change. Fold change was calculated as average relative quantitation obtained from group 1/group 2, and a value less than 1 indicates a decrease in the metabolites of group 1.

## Data Availability

The necessary data are included within the article. All data are available from the corresponding authors on reasonable request.
